# Data-driven inference for stationary jump-diffusion processes with application to membrane voltage fluctuations in pyramidal neurons

**DOI:** 10.1186/s13408-019-0074-3

**Published:** 2019-07-26

**Authors:** Alexandre Melanson, André Longtin

**Affiliations:** 1 Department of Physics, University of Ottawa, Ottawa, Canada; 2 Département de physique et d’astronomie, Université de Moncton, Moncton, Canada; 3 Centre for Neural Dynamics, University of Ottawa, Ottawa, Canada; 4 Brain and Mind Research Institute, University of Ottawa, Ottawa, Canada

**Keywords:** Stochastic differential equations, Jump-diffusion processes, Membrane noise, Channel noise, Electric fish, Pyramidal neurons, Fokker–Planck equation, Chapman–Kolmogorov equation

## Abstract

The emergent activity of biological systems can often be represented as low-dimensional, Langevin-type stochastic differential equations. In certain systems, however, large and abrupt events occur and violate the assumptions of this approach. We address this situation here by providing a novel method that reconstructs a jump-diffusion stochastic process based solely on the statistics of the original data. Our method assumes that these data are stationary, that diffusive noise is additive, and that jumps are Poisson. We use threshold-crossing of the increments to detect jumps in the time series. This is followed by an iterative scheme that compensates for the presence of diffusive fluctuations that are falsely detected as jumps. Our approach is based on probabilistic calculations associated with these fluctuations and on the use of the Fokker–Planck and the differential Chapman–Kolmogorov equations. After some validation cases, we apply this method to recordings of membrane noise in pyramidal neurons of the electrosensory lateral line lobe of weakly electric fish. These recordings display large, jump-like depolarization events that occur at random times, the biophysics of which is unknown. We find that some pyramidal cells increase their jump rate and noise intensity as the membrane potential approaches spike threshold, while their drift function and jump amplitude distribution remain unchanged. As our method is fully data-driven, it provides a valuable means to further investigate the functional role of these jump-like events without relying on unconstrained biophysical models.

## Introduction

Complex systems are ubiquitous in many areas of science, including biology, neuroscience, climatology, engineering, as well as in finance and social sciences [1, 2]. The common feature uniting such vastly different systems is the nonlinear interaction between their numerous microscopic constituents. This collective activity leads to the emergence of macroscopic order that cannot be reduced to microscopic properties [3]. It is often these macroscopic variables that can be measured experimentally, allowing the emergent dynamics of the system to be captured by low-dimensional data sets. The search for a macroscopic-level representation of the system thus relies on extracting dynamical information from observed time series [4].

For some systems, the high-dimensional, microscopic degrees of freedom can be well approximated by simple stochastic fluctuations. These fluctuations often participate as dynamical noise in the macroscopic evolution of the system. The low-dimensional representation of the system’s dynamics can then be expressed as a stochastic dynamical system [5]. More specifically, the observed data are usually assumed to satisfy a Langevin-type stochastic differential equation (SDE). A common approach is to obtain the drift and diffusion functions of this equation by estimating the first and second Kramers–Moyal coefficient [6, 7]. The resulting model is completely data-driven and captures the core phenomenology of the original data without relying on knowledge or assumptions about the microscopic constituents of the observed system. This approach has been successfully applied in a variety of contexts, ranging from neuronal dynamics [8–12], heart rate variability [13, 14], turbulence [15, 16], calibration of optical tweezers [17], and others (see Ref. [5] for a review).

In all mentioned cases, however, the noise is assumed to be purely diffusive, i.e., random fluctuations with continuous sample paths. This description is incomplete if, in addition to diffusive fluctuations, large and abrupt events appear at random times throughout the time series. In this case, jump-diffusion stochastic processes provide a more appropriate framework to model these data. Jump-diffusion processes have been used in neuroscience as a model for the spatial [18–21] and temporal [22] organization of synaptic bombardment, in physics as a model for noise-driven transport in ratchet potentials [23–25], as well as in finance [26, 27] and soil moisture dynamics [28]. The Langevin approach is likely to fail if the observed system exhibits jump-diffusion characteristics, such as skewed distributions and sudden large jumps. In such cases, and especially when the microscopic dynamics are unknown, extracting a phenomenological model from the experimental data would provide a valuable tool to probe the dynamics of the observed system, its interaction with other systems, and the interplay between diffusive and jump noise sources in shaping the observed behavior.

In this paper, we present a novel, data-driven inference method that fits a jump-diffusion SDE to experimental time series. By detecting jumps through threshold-crossing and by calculating the contribution of diffusive fluctuations that are falsely detected as jumps, we iteratively estimate the drift function, noise intensity, jump rate, and jump amplitude distribution. The result of this semi-parametric method is a jump-diffusion SDE that successfully fits the original data. Our method is applicable in cases where these data are stationary, with additive diffusive noise and Poisson jumps. Note that other studies have attempted to infer jump-diffusion dynamics from data, but they rely on assuming a parametric form of the jump amplitude distribution [29–31], or consider only Lévy processes [32, 33].

We test our method with two validation cases, where realizations of known jump-diffusion processes with different characteristics are used as validation data. In both cases, by using only the simulated time series, we precisely recover the correct parameters and functions used in the original simulations. We also compare the autocorrelation functions (ACF) of the validation data and of fitted SDE. We then apply our method to recordings of intrinsic membrane voltage fluctuations in pyramidal neurons of electric fish. These recordings contain sudden and unpredictable jump-like events that occur among more typical diffusive membrane noise. Although the exact biophysical origin of these fluctuations is unknown, we find that the recordings are well fitted by jump-diffusion processes. We evaluate goodness of fit quantitatively by comparing the observed and the estimated probability density functions (PDF) and power spectral densities (PSD). Interestingly, we find that some pyramidal cells increase their jump rate and noise intensity as the membrane potential approaches spike threshold, while their drift function and jump amplitude distribution remain unchanged.

In Sect. 2, we present the various steps involved in the inference procedure, including the detection scheme, the calculation relating to false positive, and the iterative approach. In Sect. 3, we validate this procedure against one pure diffusion and two jump-diffusion test cases, and then we apply it to neurophysiological recordings of membrane noise. This is followed by a discussion on the possible generalizations of our method and on future work with the experimental data (Sect. 4).

## Methods

### Definitions and overview

Let $\{X(t)\}$ represent data in the form of a stationary time series. In what follows, $\{X(t)\}$ is obtained either from experimental observations or from numerical simulation. In either case, the situation of interest here is when $\{X(t)\}$ exhibits both diffusive fluctuations and abrupt events (henceforth called jumps). From $\{X(t)\}$, our goal is then to fit a jump-diffusion SDE of the form

1 $$ dY(t) = F\bigl(Y(t)\bigr)\,dt + \sqrt{2D} \,dW(t) + dJ(t), $$

where *F* is the drift function, *D* is the noise intensity, and $W(t)$ is a Wiener process (i.e., Brownian motion). Here $J(t)$ is a compound Poisson process representing the jumps

2 $$ J(t) = \sum_{i=0}^{N_{\lambda }(t)} B_{i}, $$

where $N_{\lambda }(t)$ is a Poisson point process with rate *λ*, and the ${B_{i}}$’s are the independent and identically distributed jump amplitudes drawn from a distribution $Q_{B}$. For a small enough sampling interval, or time step Δ*t*, jumps will occur with probability $\varGamma _{B} \equiv \lambda \Delta t$.

Although the method developed herein is applicable to a wide array of experimental data types, we do, however, impose certain conditions on the underlying dynamical process. Notably, we limit our analysis to systems where the dynamics are stationary (in the strict sense), the diffusion noise is additive, the jumps have positive amplitudes ($B_{i}>0$), and where the Poisson rate *λ* is constant in time and small enough so that $\varGamma _{B} \ll 1$. Furthermore, we assume here that *F* is continuous and that a single stable fixed point arises from the deterministic part of the dynamics, but our method could be generalized to multistable systems. Except for this restriction, we assume no particular shape for the drift function as long as it generates a stationary process. Finally, we assume that, on average, jump amplitudes are greater than the typical magnitude of diffusive increments: $\mathrm{E}[B_{i}] > (2D\Delta t)^{\frac{1}{2}}$ by one order of magnitude of more.

In terms of the time series itself, we assume that the data were sampled at a high-frequency, such that Δ*t* can be assumed to be small with respect to the total duration of the time series. Note that the value of Δ*t* is set from the experiments that produced the data, and so is not a variable we can control. For experimental data, however, it is possible that the Markov property breaks down on the timescale of individual observations [5], but we assume that, in that case, the time series can be downsampled to a timescale where the Markov property then holds.

Furthermore, in the cases considered below, jumps appear unambiguously in the data, and so a jump-diffusion approach is warranted. In situations where this is not the case, the presence of jumps can be assessed from the fourth-order Kramers–Moyal coefficient, which is non-vanishing for processes with discontinuities [31, 34]. Lastly, note that we make no assumptions regarding the structure of the ACF of $\{X(t)\}$; we only assume stationarity in these data. In fact, we compare the ACFs (and PSDs) of time series generated by the fitted SDE with those of the original data as a means of validating the inference procedure.

Below we develop a data-driven inference procedure that successfully generates the estimates *D̂*, *λ̂*, *F̂*, and $\hat{Q}_{B}$, where the unknown functions *F* and $Q_{B}$ are estimated non-parametrically. This inference procedure results in a fitted stochastic process $Y(t)$ that is an adequate model of the original data. In this sense, we implicitly assume that the data $\{X(t)\}$ are sampled from a realization of $Y(t)$. In our calculations, we thus associate the first-order equilibrium PDF of $Y(t)$, $P_{Y}$, to the empirical PDF of $\{X(t)\}$ (obtained by kernel density estimation) $P_{X}$.

Our approach is predicated on the detection of jumps in the data via the application of a threshold $\theta ^{*}>0$ on the increments $\Delta X(t+\Delta t) \equiv X(t + \Delta t) - X(t)$. This procedure creates a pool of detected jumps with various amplitudes. Let $Q_{C}$ be the empirical PDF estimated from these measured jump amplitudes. Also, let *n* be the total number of increments in the time series, and *m* be the number of increments whose value is greater than $\theta ^{*}$. We define the (overall) jump detection probability as $\varGamma _{C} \equiv \operatorname{Prob} \{ \text{detecting an increment larger than }\theta ^{*}\text{ across an interval }\Delta t \}$, which we estimate from the data as $\varGamma _{C} = \frac{m}{n}$.

**Figure 1:**
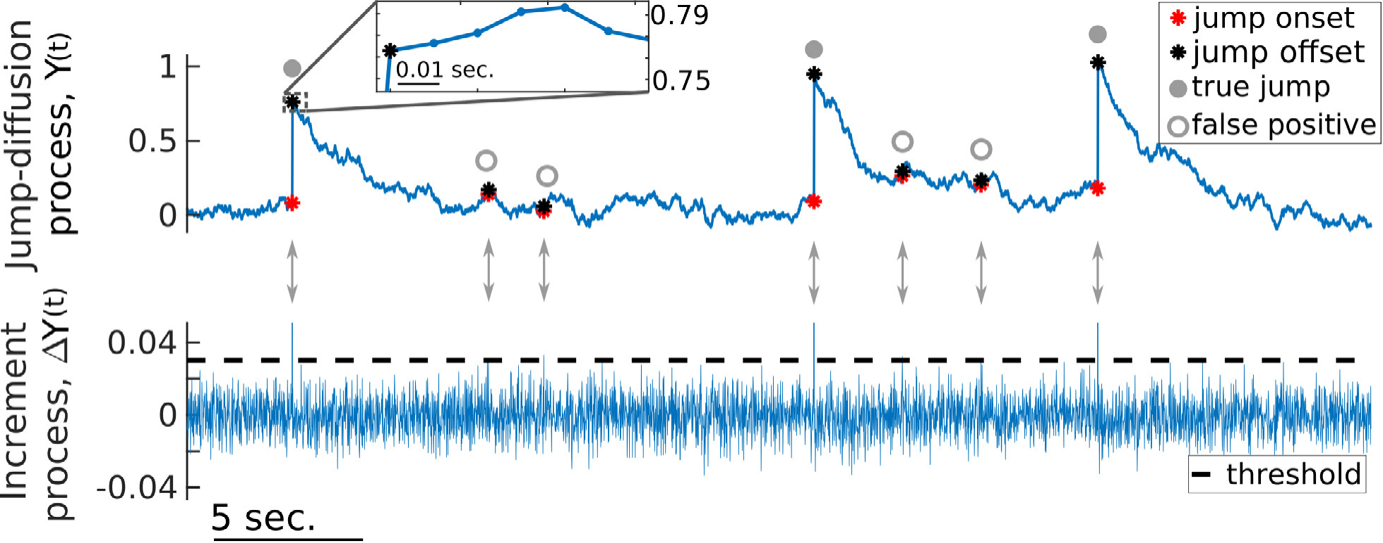
*Jumps are detected by applying a threshold on the increments*, *but this also creates false positives*. *Top*: An example of a simulated jump-diffusion process where both true jumps (grey dots) and false positives (grey rings) are detected. The inset shows how the jump offset can be registered even if subsequent increments are positive. *Bottom*: Increment time series of the simulated process in the top panel. A jump is detected every time an increment exceeds the threshold. In this exaggerated case, true jumps are well above threshold, while false positives barely exceed it. This clear separation is not generally the case

An inherent challenge with this threshold-crossing approach is that, in addition to the true jumps generated by the compound Poisson process, we also unavoidably detect large diffusive fluctuations that are falsely identified as jumps, henceforth called false positives (FP; Fig. 1, top). A direct estimation of the true jump rate *λ* and of the true jump amplitude distribution $Q_{B}$ is thus impossible because the detected jump pool consists of a mixture of true jumps and false positives. Our main contribution, and the central component of our inference procedure, is the calculation of FP-related statistics, namely the FP detection probability $\varGamma _{A}$ ≡ Prob{detecting a diffusive increment larger than $\theta ^{*}$ across a given time step Δ*t*} and the distribution from which FP amplitudes are drawn $Q_{A}$. Once $\varGamma _{A}$ and $Q_{A}$ are calculated, we then extract *λ* (or, equivalently, $\varGamma _{B}$) and $Q_{B}$ from $\varGamma _{C}$ and $Q_{C}$. Note that the subscripts “A”, “B”, and “C” will hereafter refer to FPs, true jumps, and both combined, respectively. More precisely, we measure quantities in “C” from the detected jump pool, we calculate FP statistics in “A”, and we seek the true jump statistics in “B”.

### Choice of threshold

To ensure that a minimal number of true jumps are missed during the jump detection procedure, the threshold $\theta ^{*}$ should ideally be set as low as possible. If set too low, however, the number of FPs becomes so large that the statistics of true jumps, i.e., *λ* and $Q_{B}$, cannot be extracted from the statistical fluctuations of $Q_{C}$ and $\varGamma _{C}$. We thus aim for an intermediate value of $\theta ^{*}$ that captures most true jumps while allowing a manageable number of FPs. This is done by relying on our assumption of positive jump amplitudes and, more precisely, by exploiting the asymmetry between positive and negative increment statistics. Note that the threshold depends implicitly on the time step of the original time series: a smaller Δ*t* means that diffusive fluctuations are smaller, and thus a smaller value of $\theta ^{*}$ can be used.

**Figure 2:**
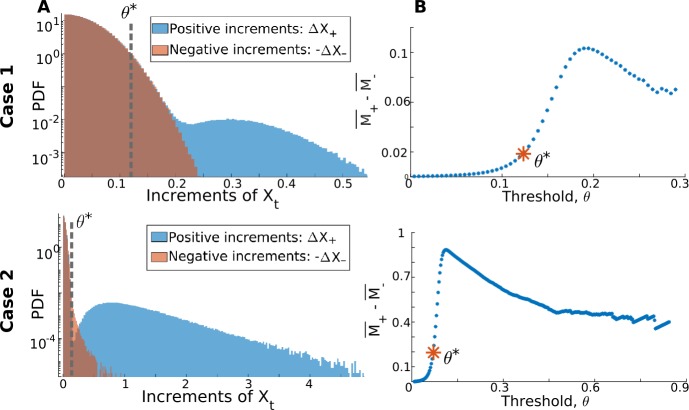
*The choice of* $\theta ^{*}$ *is based on comparing the statistics of positive and negative increments*. We show here how this strategy is applied to the two jump-diffusion validation cases presented in Sect. 3.2. (**A**) The presence of true jumps allows the PDF of positive increments (blue histogram) to be differentiated from that of negative increments (red histogram) above a certain threshold (dashed line). (**B**) This threshold is chosen as the inflection point (red asterisks) of the difference between the sample means of the (truncated) positive and negative increments

Let $\{\Delta X_{+}\}$ and $\{\Delta X_{-}\}$ be the sets of positive and negative increments of $\{X(t)\}$, respectively (Fig. 2(A)). In addition, let $M_{+}(\theta ) = \{\Delta X _{+}: \Delta X_{+} > \theta \}$ and $M_{-}(\theta ) = \{-\Delta X_{-}: -\Delta X_{-} > \theta \}$ be the reduced sets truncated by *θ*, where *θ* spans the common range of $\{\Delta X_{+} \}$ and $\{-\Delta X_{-}\}$. We use the difference between the sample mean of these sets $\overline{M_{+}} - \overline{M_{-}}$ as a function of *θ* to quantify the relative importance of true jumps with respect to diffusive fluctuations, over different increment sizes. The value of *θ* for which $\overline{M_{+}} - \overline{M_{-}}$ is a maximum corresponds to the greatest separability between positive and negative increments. We find, however, that using the inflection point, i.e., where the second derivative becomes 0, located to the left of this maximum (Fig. 2(B), asterisks) is a better choice of threshold. This slightly lower value retains a greater range of true jumps, which is desirable, while avoiding the inclusion of an overwhelmingly large number of FPs. Choosing this inflection point rather than the maximum impacts primarily the estimation of *λ*, since it relies on the proper detection of true jumps in the time series. A slightly higher value of $\theta ^{*}$ will introduce a bias in *λ̂*. For instance, in the first jump-diffusion validation case that we consider in Sect. 3.2, choosing the maximum as the threshold roughly yields a 1% increase in the error on *λ̂* compared to when the inflection point is used. Our approach for setting $\theta ^{*}$ is thus motivated by the fact that it is advantageous to choose a value as small as possible for $\theta ^{*}$, and the inflection point in the curve of $\overline{M_{+}} - \overline{M_{-}}$ provides a reliable way to achieve this.

### Jump detection

Here we describe how the threshold is applied to the increments in order to generate the detected jump pool. We apply a detection scheme tailored specifically to handle two aspects that we observe in experimental data with jumps, and it is inspired by the method used in [35]. Firstly, if the data are resolved on a fine enough time scale, jumps may last longer than a single sampling interval. Secondly, jumps need not be followed immediately by negative increments. In data, and in some simulations as well, the diffusive increments following a jump can still be positive, but we seek a method for identifying when the jump actually ends (Fig. 1, inset). These two considerations shape the method used to calculate the FP amplitude distribution in Sect. 2.4.2.

When a given increment is larger than the threshold, a jump onset time $T_{\mathrm{on}}$ is registered, and if the next increment is below threshold, the associated offset time $T_{\mathrm{off}}$ is registered (even if this increment is positive). This defines a jump of duration $T_{\mathrm{off}}-T_{\mathrm{on}} = \Delta t$, where Δ*t* is the sampling interval of the data. We henceforth refer to this type of jump as a singlet, as it spans the duration of a single time step. In contrast, if two or more successive increments are above threshold, then the jump is of duration $2\Delta t$ or more, which we refer to as a doublet, triplet, and so forth. In other words, the jump offset time is only registered at the end of the sequence of above-threshold increments. With the onset and offset times identified, a jump amplitude is defined as the difference between $X(T_{\mathrm{off}})$ and $X(T_{\mathrm{on}})$.

### FP and true jump statistics

Here we present the calculations of the FP detection probability $\varGamma _{A}$ and of the FP amplitude distribution $Q_{A}$. We then show how these quantities are used to extract the true jump rate *λ* and the true jump amplitude distribution $Q_{B}$ from the detected jump probability $\varGamma _{C}$ and the detected jump amplitude distribution $Q_{C}$. For the calculations in this subsection, we assume that the drift function *F* and noise intensity *D* are known. In the next subsection, we show how these calculations can be incorporated into an iterative scheme that allows the simultaneous estimation of all unknowns, including *F* and *D*. Furthermore, note that the FP-related calculations involve only the diffusive part of Eq. (1) since, by definition, FPs occur during the purely diffusive segments between true jumps. The calculations in Sects. 2.4.1 and 2.4.2 thus pertain only to diffusive increments $\Delta Y^{\mathrm{diff}}(t)$. Finally, note that we perform validation tests of the calculations of $\varGamma _{A}$ and $Q_{A}$ in Sect. 3.1.

#### FP detection probability

As mentioned in Sect. 2.1, sampling a jump-diffusion process such as Eq. (1) at finite intervals and applying a threshold on the observed increments leads to the detection of FPs, i.e., diffusive (rather than true jump) increments larger than the threshold. Importantly, these FPs occur with a probability that depends on the value of the process at the start of the interval. Let this conditional detection probability be defined as

3 $$\begin{aligned} \alpha (y) &\equiv \operatorname{Prob}\bigl\{ \text{detecting an FP in the interval } [t,t+\Delta t] \text{, given that } Y(t)=y \bigr\} \\ &= \operatorname{Prob}\bigl( \Delta Y^{\mathrm{diff}}(t+\Delta t) > \theta ^{*} | Y(t)=y\bigr). \end{aligned}$$

As *α* does not depend explicitly on time, this definition relies on our assumption that $Y(t)$ is stationary. The *y*-dependence arises from the drift function *F*. Indeed, if the drift function is positive (respectively, negative) at a given time, it biases diffusive fluctuations toward (away from) the threshold. This translates into an FP detection probability that assumes higher values when $F(y)>0$ than when $F(y)<0$. We now turn to the explicit calculation of $\alpha (y)$.

Let $\varXi _{\Delta Y|Y}(\xi | Y(t)=y)$ denote the PDF of $\Delta Y^{\mathrm{diff}}(t+\Delta t)$ conditioned on the value of the process at the start of the interval, and where *ξ* assumes the possible values of the increments. Note that, because the time step remains constant, it is always implied that the increments are defined across an interval Δ*t*. Given that we assume Δ*t* to be sufficiently small, we approximate $\varXi _{\Delta Y|Y}$ as the short-time propagator of the Fokker–Planck equation [36, 37] associated with the diffusive part of jump-diffusion process (recall that what concerns us here are the purely diffusive segments between the true jumps of $Y(t)$). We thus have $\Delta Y^{\mathrm{diff}}(t+\Delta t) \approx {\mathcal{N}} (F(Y(t))\Delta t,2D\Delta t)$ and

4 $$ \varXi _{\Delta Y|Y}\bigl(\xi |Y(t)=y\bigr) \approx \frac{1}{\sqrt{4 \pi D \Delta t}} \exp \biggl(- \frac{ (\xi - F(y) \Delta t )^{2}}{4 D \Delta t} \biggr), $$

that is, a Gaussian distribution with mean $F(y)\Delta t$ and variance $2D\Delta t$.

For the test cases presented in Sect. 3 (with $\Delta t = 10^{-4}\text{ s}$), we have validated this approximation by comparing it with numerical solutions of the associated Fokker–Planck equation solved at a finer temporal resolution ($\Delta t/1000$) across the time step Δ*t*. The numerical solutions were indeed well fitted with the approximation in Eq. (4) (not shown). Numerical integration was performed with a custom partial differential equation solver that implements a finite volume discretization along with the fully implicit Euler scheme. The advective term was treated with the upwind scheme, and a linear interpolation profile for the spatial derivative was applied to the diffusive term. The resulting algebraic equation was solved with the tridiagonal matrix algorithm [38].

Once the conditional PDF of the increments is evaluated with Eq. (4), we calculate the conditional FP detection probability, given that the process starts at *y*, as follows:

5 $$ \alpha (y) = \int _{\theta ^{*}}^{\infty }\varXi _{\Delta Y|Y}\bigl(\xi |Y(t)=y\bigr) \,\mathrm{d}\xi , $$

that is, the probability of observing an increment larger than $\theta ^{*}$ starting at *y*. Finally, the unconditional FP detection probability is calculated based on the empirical PDF of $\{X(t)\}$, $P_{X}$:

6 $$ \varGamma _{A} = \int _{-\infty }^{\infty }\alpha (y)P_{X}(y)\, \mathrm{d}y. $$

We validate these calculations in Sect. 3.1.

#### FP amplitude distribution

**Figure 3:**
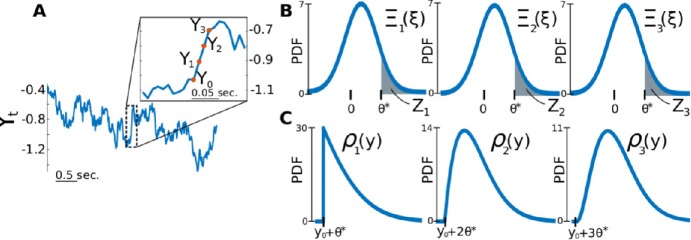
*The estimate of* $Q_{A}$ *is obtained through a probabilistic analysis of FP detection*. (**A**) Example of a diffusive fluctuation registered as an FP triplet. (**B**) The situation in A is addressed by calculating $\rho _{i}$, the PDF of $Y_{i}$ conditioned on $Y_{0}$. (**C**) From $\rho _{i-1}$, we then calculate $\varXi _{i}$, the PDF of $\Delta Y_{i}$ conditioned on $Y_{0}$, which is used to evaluate $Z_{i}$ (shaded area), the probability that the *i*th increment is above threshold, given $Y_{0}$. Note that, although they look similar, the $\varXi _{i}$’s are slightly different from each other

We now proceed with the calculation of $Q_{A}$, i.e., the distribution from which FP amplitudes are drawn. First, recall that our detection scheme allows for jumps of different durations (Sect. 2.3). As such, the detection of an FP implies either a succession of above-threshold increments (e.g., Fig. 3(A)) or, at least, a single above-threshold increment. Let $T^{\mathrm{FP}}_{\mathrm{on}}$ denote the FP onset time, that is, the time at the start of the first above-threshold increment, and let $Y_{0} \equiv Y(T^{\mathrm{FP}}_{\mathrm{on}})$ be referred to as the starting value of the FP. Moreover, let *τ* denote the FP duration, an integer multiple of Δ*t*, such that $\tau = \Delta t$ corresponds to an FP singlet, $\tau = 2\Delta t$ to an FP doublet, and so forth.

In order to calculate $Q_{A}$, let us first identify the factors that influence FP amplitudes. Firstly, it must be noted that the FP amplitudes will exhibit a similar *y*-dependence as that discussed in the preceding section. Indeed, an increment starting at $Y_{0}\equiv Y(T^{\mathrm{FP}}_{\mathrm{on}})=y$ will tend to be larger when $F(y)>0$ than when $F(y)<0$. Secondly, the amplitude of an FP will also depend on its duration, *τ*. For instance, the three increments of a triplet FP will summate and tend to have a larger amplitude than that of a singlet. The FP amplitudes *A* will thus covary with $Y_{0}$ and with *τ*, but note that *τ* also depends on $Y_{0}$. Indeed, longer FPs will tend to occur where the drift function is more positive, and vice versa. To account for these dependencies, let us define the trivariate random variable $\{A, \tau , Y_{0} \}$, distributed according to its joint PDF $P_{A,\tau ,Y_{0}}(a,i\Delta t,y)$, where we explicitly write *τ* as an integer multiple of Δ*t*. What we seek then is the marginal:

7 $$ Q_{A}(a) = \int _{-\infty }^{\infty }\sum_{i=1}^{\infty }P_{A,\tau ,Y _{0}}(a,i \Delta t,y) \,\mathrm{d}y, $$

where the sum extends over all possible FP durations, and where $a>0$ represents all possible amplitudes. From the definition of conditional PDFs, we can expand the joint PDF as follows:

8 $$\begin{aligned} P_{A,\tau ,Y_{0}}(a,i\Delta t,y) &= P_{A|\tau ,Y_{0}}(a|i\Delta t,y)P _{\tau ,Y_{0}}(i\Delta t,y) \\ &=P_{A|\tau ,Y_{0}}(a|i\Delta t,y)P_{\tau |Y_{0}}(i\Delta t|y)P_{Y _{0}}(y), \end{aligned}$$

where $P_{\tau |Y_{0}}(i\Delta t|y)=\operatorname{Prob} (\text{detecting an FP of duration } i\Delta t\text{, given the starting value } y )$ is the conditional probability mass function of the FP duration *τ*. We can thus write Eq. (7) as^1^

9 $$ Q_{A}(a) = \int _{-\infty }^{\infty } \Biggl( \sum _{i=1}^{\infty }P_{A| \tau ,Y_{0}}(a|i\Delta t,y) P_{\tau |Y_{0}}(i\Delta t|y) \Biggr) P _{Y_{0}}(y) \,\mathrm{d}y. $$

The sum in the large parentheses is a function of *y*, and Eq. (9) is merely calculating its average with respect to the starting value $Y_{0}$. This sum can further be interpreted as a so-called mixture distribution: consider a collection of random variables, one of which is chosen according to a certain probability (its *mixture weight*) and is then realized according to its own PDF (its *mixture component*). The outcome of this experiment is itself a random variable whose PDF is called a *mixture distribution* and is expressed as a sum over the PDFs of the random variables in the collection, weighted by their respective probabilities. In our case, for a fixed value of $Y_{0}$, an FP duration is drawn according to a countable set of mixture weights $P_{\tau |Y_{0}}$, and an FP amplitude is then realized according to the associated mixture component $P_{A|\tau ,Y_{0}}$. In practice, the sum will be truncated after the first few terms because the subsequent mixture weights become negligible.

From Eq. (9), we see that in order to arrive at the desired $Q_{A}$, the functions $P_{A|\tau ,Y_{0}}$, $P_{\tau |Y_{0}}$, and $P_{Y_{0}}$ must first be calculated. Let us first consider the latter. Because $Y_{0}$ represents, by definition, the value of $Y(t)$ at the start of an above-threshold increment, we can express its PDF in terms of the joint PDF of $\Delta Y^{\mathrm{diff}}(t+\Delta t)$ and $Y(t)$:

10 $$\begin{aligned} P_{Y_{0}}(y) &= K \int _{\theta ^{*}}^{\infty }P_{\Delta Y,Y}(\xi ,y) \, \mathrm{d} \xi \\ &= K \int _{\theta ^{*}}^{\infty }\varXi _{\Delta Y|Y}\bigl(\xi |Y(t)=y\bigr) P_{Y}(y) \,\mathrm{d}\xi \\ &= K P_{Y}(y) \int _{\theta ^{*}}^{\infty }\varXi _{\Delta Y|Y}\bigl(\xi |Y(t)=y\bigr) \,\mathrm{d}\xi \\ &= K P_{X}(y) \alpha (y), \end{aligned}$$

where *K* is a normalization constant and where, in the last line, we have replaced $P_{Y}$ by the empirical PDF of $\{X(t)\}$. Note that we integrate with $\theta ^{*}$ as a lower bound in order to enforce that $Y_{0}$ is associated with the onset of an above-threshold increment. Let us now consider the calculation of $P_{A|\tau ,Y_{0}}$.

In what follows, we simplify the notation by labeling time with the index *i*, such that $i=0$ represents the time $T^{\mathrm{FP}}_{\mathrm{on}}$, $i=1$ the time $T^{\mathrm{FP}}_{\mathrm{on}}+\Delta t$, $i=2$ the time $T^{\mathrm{FP}}_{\mathrm{on}} + 2\Delta t$, and so forth. With this notation, $Y_{i} \equiv Y(T^{\mathrm{FP}}_{\mathrm{on}} + i\Delta t)$ denotes the *i*th point following the FP onset time, and $\Delta Y_{i}^{\mathrm{diff}} \equiv Y_{i} - Y_{(i-1)} $ the *i*th diffusive increment. Furthermore, our focus here is on FPs of duration, say $i\Delta t$, which corresponds to a sequence of *i* successive above-threshold increments. As such, the forthcoming calculations involve PDFs that are implicitly conditioned on the event $\{\Delta Y^{\mathrm{diff}}_{n} > \theta ^{*}, \forall n\le i\}$.

For an FP of duration $\tau = i\Delta t$ starting at $Y_{0}$, we define its amplitude as $A = Y_{i} - Y_{0}$, and we seek the conditional PDF $P_{A|\tau ,Y_{0}}$. For this purpose, let $\rho _{i}(y)\equiv P_{Y _{i}|Y_{0}}(y|y_{0})$ denote the PDF of $Y_{i}$, $i>1$, conditioned on $Y_{0}$. Since *A* is expressed as the difference between $Y_{i}$ and $Y_{0}$, we can directly write

11 $$ P_{A|\tau ,Y_{0}}(a|i\Delta t,y_{0}) = \rho _{i}(a+y_{0}). $$

The $\rho _{i}$’s, for $i>1$, are evaluated sequentially based on the fact that $Y_{i}=\Delta Y^{\mathrm{diff}}_{i} + Y _{i-1}$. The PDF of this sum, conditioned on $Y_{0}$, gives

12 $$\begin{aligned} \rho _{i}(y) &= \int _{-\infty }^{\infty }P_{\Delta Y_{i},Y_{i-1}|Y_{0}}( \xi ,y-\xi |y_{0})\,\mathrm{d}\xi \\ &= \int _{-\infty }^{\infty }P_{\Delta Y_{i}|Y_{i-1},Y_{0}}(\xi |y- \xi ,y_{0})P_{Y_{i-1}|Y_{0}}(y-\xi |y_{0})\,\mathrm{d}\xi \\ &= \int _{-\infty }^{\infty }P_{\Delta Y_{i}|Y_{i-1}}(\xi |y-\xi ) \rho _{i-1}(y-\xi )\,\mathrm{d}\xi . \end{aligned}$$

To enforce the condition of above-threshold increments, $\{ \Delta Y^{\mathrm{diff}}_{n} > \theta ^{*}, \forall n\le i\}$, we evaluate $P_{\Delta Y_{i}|Y_{i-1}}$ based on Eq. (4), but we truncate the distribution below $\xi =\theta ^{*}$:

13 $$ P_{\Delta Y_{i}|Y_{i-1}}(\xi |y) = K \textstyle\begin{cases} \varXi _{\Delta Y_{i}|Y_{i-1}} (\xi |Y(t)=y) & \text{if } \xi > \theta ^{*}, \\ 0 & \text{otherwise}, \end{cases} $$

where *K* is a normalization constant. With Eq. (13) and (12), we finalize the calculation of $P_{A|\tau ,Y_{0}}$ in Eq. (11). In Fig. 3(B), we see the representation of the $\rho _{i}$ for an FP triplet. From $\rho _{i}$, we can also calculate the PDF of $\Delta Y^{\mathrm{diff}}_{i}$, conditioned on $Y_{0}$ (this will be useful in the calculation of $P_{\tau |Y_{0}}$). Let this PDF be defined as $\varXi _{i}(\xi ) \equiv P_{\Delta Y_{i}|Y_{0}}(\xi | y_{0})$. We calculate it as a marginal over $Y_{i-1}$:

14 $$\begin{aligned} \varXi _{i}(\xi ) &= \int _{-\infty }^{\infty }P_{\Delta Y_{i}, Y_{i-1}|Y _{0}}(\xi ,y|y_{0}) \,\mathrm{d}y \\ &= \int _{-\infty }^{\infty }\varXi _{\Delta Y_{i}| Y_{i-1}}(\xi |y) \rho _{i-1}(y) \,\mathrm{d}y, \end{aligned}$$

where the dependence of $\varXi _{\Delta Y_{i}|Y_{i-1}}$ on $Y_{0}$ disappears because of the Markov property. In Fig. 3(C), we see the representation of the $\varXi _{i}$’s for an FP triplet.

We now turn to the calculation of $P_{\tau |Y_{0}}$, the probability of observing an FP of duration *τ*, conditioned on the starting value $Y_{0}$. We are interested in the conditional probability of the event $\{\tau = i\Delta t\}$, where *i* is an integer. This event is equivalent to the intersection of the events $E_{1} \equiv \{\Delta Y _{1}^{\mathrm{diff}} > \theta ^{*}\}, E_{2} \equiv \{\Delta Y_{2}^{\mathrm{diff}} > \theta ^{*}\},\ldots$ and $E_{i+1}' \equiv \{\Delta Y_{i+1}^{\mathrm{diff}}\le \theta ^{*}\}$. In other words, we obtain an FP of duration $i \Delta t$ when the first *i* increments are above threshold, but the $(i+1)$th increment is below threshold.

By successively applying the definition of conditional probability, we can expand $P_{\tau |Y_{0}}$ as follows:

$$\begin{aligned} P_{\tau |Y_{0}}(i\Delta t|y_{0}) & = \operatorname{Prob}(\tau = i\Delta t | y_{0}) \\ & = \operatorname{Prob} \Biggl[E'_{i+1} \cap \Biggl( \bigcap_{n=1}^{i} E _{n} \Biggr) \Big| y_{0} \Biggr] \\ & = \operatorname{Prob} \Biggl[E'_{i+1} \Big| \Biggl( \bigcap_{n=1}^{i} E _{n} \Biggr), y_{0} \Biggr] \cdot \operatorname{Prob} \Biggl[E_{i} \Big| \Biggl( \bigcap_{n=1}^{i-1} E_{n} \Biggr), y_{0} \Biggr] \\ &\quad {}\cdot \operatorname{Prob} \Biggl[E_{i-1} \Big| \Biggl( \bigcap_{n=1}^{i-2} E_{n} \Biggr), y_{0} \Biggr]\cdot \ldots \cdot \operatorname{Prob} (E _{2} | E_{1}, y_{0} ) \cdot \operatorname{Prob} (E_{1} | y_{0} ). \end{aligned}$$

Let $Z_{i}(y_{0})\equiv \operatorname{Prob} [E_{i} | ( \bigcap_{n=1}^{i-1} E_{n} ), y_{0} ]$, $i>1$, represent the probability that the *i*th increment is above threshold, given that the $i-1$ previous increments were also above threshold, and given the starting value $y_{0}$. These $Z_{i}$’s can be calculated from the $\varXi _{i}$’s of Eq. (14) as (Fig. 3(B), shaded area):

15 $$\begin{aligned} Z_{i}(y_{0}) & = \operatorname{Prob} \bigl(\Delta Y^{\mathrm{diff}}_{i} > \theta ^{*} | \Delta Y^{\mathrm{diff}}_{n} > \theta ^{*}, \forall n< i; y_{0} \bigr) \\ & = \int _{\theta ^{*}}^{\infty }\varXi _{i}(\xi )\, \mathrm{d}\xi . \end{aligned}$$

We now arrive at the desired probability mass function:

16 $$ P_{\tau |Y_{0}}(i\Delta t|y_{0}) = \operatorname{Prob}(\tau = i\Delta t | y _{0}) = \bigl(1-Z_{i+1}(y_{0}) \bigr) \prod _{n=1}^{i} Z_{n}(y_{0}), $$

where we have used the fact that $1-Z_{i+1}$ is equal to the probability that the $(i+1)$th increment is below threshold, and where we have defined $Z_{1}(y_{0}) \equiv \operatorname{Prob} (E_{1} | y_{0} )= \alpha (y_{0})$, i.e., the probability that the first increment after $Y_{0}$ is above threshold. Once Eq. (10), (11), and (16) are evaluated, we apply Eq. (9) to obtain the desired $Q_{A}$. Using this approach, we obtain an excellent agreement between theory and simulations, as reported in Sect. 3.1.

#### True jump rate

Our estimate of the true jump rate *λ* relies on the knowledge of the overall jump detection probability $\varGamma _{C}$ and on the FP detection probability $\varGamma _{A}$ (both defined in Sect. 2.1). Recall that we calculate $\varGamma _{A}$ from Eq. (6), while we estimate $\varGamma _{C}$ directly from the data as $m/n$, where *m* is the number of time steps with $\Delta X(t) > \theta ^{*}$ (either from a true jump or an FP) and *n* is the total number of time steps in the data time series. On the other hand, from the definition of $\varGamma _{C}$ we can write:

17 $$\begin{aligned} \varGamma _{C} &\equiv \operatorname{Prob} \bigl\{ \text{detecting an increment larger than }\theta ^{*}\text{ across an interval }\Delta t \bigr\} \\ &= \operatorname{Prob} \bigl\{ (\text{detecting an FP across }\Delta t)\cup ( \text{detecting a true jump across }\Delta t) \bigr\} \\ & = \varGamma _{A} + \varGamma _{B} - \varGamma _{A} \varGamma _{B} \\ &= \varGamma _{A} + \lambda \Delta t - \varGamma _{A} \lambda \Delta t, \end{aligned}$$

where $\varGamma _{B} = \lambda \Delta t$ is the probability of observing a true jump in an interval Δ*t*. This is merely a statement of the addition law of probability, which would read: the probability of detecting a jump in an interval Δ*t* is the sum of the probability of observing a true jump, plus that of observing an FP, minus the probability of observing both at the same time, where we use the fact that FPs and true jumps are independent events. For the test cases described in Sect. 3.2, isolating *λ* in Eq. (17) is accurate up to an error of 0.02%.

#### True jump amplitude distribution

As in the previous subsection, we obtain an estimate for the true jump amplitude distribution $Q_{B}$ based on the empirical PDF of jump amplitudes measured from the time series $Q_{C}$ and on the calculated FP amplitude distribution $Q_{A}$. Because detected jumps are a mixture of true jumps and FPs, we can write, a priori,

18 $$ Q_{C} = W_{A} Q_{A} + W_{B}Q_{B}, $$

where $W_{A} = \frac{\varGamma _{A}}{\varGamma _{C}}$ is the probability that a detected jump is an FP, and $W_{B}= \frac{\varGamma _{B}}{\varGamma _{C}}$ that it is a true jump. The subtlety here is that, contrary to FPs, true jumps are never detected on their own, as they always summate with a diffusive fluctuation. In other words, we never observe the $B_{i}$’s directly, but rather the $B_{i}$’s plus a diffusive increment. Over a short enough time step, diffusive increments are Gaussian variables and are approximately independent of each other. For the purpose of calculating $Q_{B}$, we will thus assume these increments are Gaussian with mean zero and variance $2D \Delta t$. Properly accounting for the *y*-dependence of the mean would be more precise, but would require $Q_{C}$ to be broken down into a family of distributions parameterized by *y*, which would require a very large number of detected jumps in the data.

Let *Ξ̃* represent a Gaussian distribution with zero mean and variance $2D\Delta t$. The $Q_{B}$ in Eq. (18) should thus be replaced by the convolution $\tilde{\varXi }\ast Q_{B}$. Furthermore, $W_{A}$ must in fact be reduced by a factor $(1-\varGamma _{B})$ to account for the probability that FPs can occur during the same interval as a true jump. This leads to

19 $$ Q_{C} = \frac{\varGamma _{A} (1 - \varGamma _{B})}{\varGamma _{C}} Q_{A} + \frac{ \varGamma _{B}}{\varGamma _{C}} (\tilde{\varXi }\ast Q_{B}). $$

From this equation, we isolate the convolution term and apply the basic deconvolution algorithm [39] to extract $Q_{B}$. Let *f* be a measured, convolved signal, where the convolution kernel *h* is known. We seek the intact signal *g*, such that $f = (h \ast g)$. We compute an estimate of *g* at each iteration as $g_{k+1} = g_{k} + [f - (h \ast g_{k})]$, with $g_{0} = f$. The algorithm converges once the correct signal is reached, since the residual between *f* and $(h \ast g_{k})$ then becomes zero.

### Iterative procedure, noise intensity, and drift function

**Figure 4:**
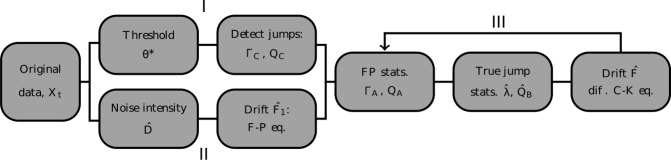
*Overview of the flow of our iterative procedure*. In both validation cases, a satisfying estimate is obtained after about 10 iterations. The threshold and noise intensity are estimated directly from the data in I and II, while the true jump rate and amplitude distribution, as well as the drift function, appear in the iterative phase III. Note that the true jumps statistics are not yet established in II, and this is why we resort to the Fokker–Planck equation as a means to obtain a preliminary guess of the drift function $\hat{F}_{1}$. A more refined estimate of *F* is later obtained at the end of branch III

We now turn to the problem of the simultaneous data-driven estimation of all the unknowns in Eq. (1). To this end, we incorporate the calculations of Sect. 2.4 in the iterative scheme depicted in Fig. 4, which consists of three main branches. The first two initial branches, I and II, are independent and are performed only once; this is followed by branch III where the iterations take place. In branch I, the threshold is set (Sect. 2.2) and then applied to the time series to yield the detected jump pool, from which $\varGamma _{C}$ and $Q_{C}$ are obtained (Sect. 2.3). The noise intensity is estimated in branch II, along with an initial guess for the drift function $\hat{F}_{1}$, which are both used in the first iteration of branch III to calculate $\hat{\varGamma }_{B}$ and *λ̂* (Sect. 2.4). The last step in branch III uses *D̂*, $\hat{\varGamma }_{B}$, and *λ̂* to estimate the drift function *F̂*, which is fed back to the first step of branch III in order to iteratively refine the estimation procedure.

#### Noise intensity

As depicted in Fig. 4, the estimation of *D* does not rely on the value chosen for $\theta ^{*}$. It does, however, still use the notion of applying a threshold on the increments. Indeed, calculating *D̂* relies on partitioning $\{X(t)\}$ into mostly jump-free segments, the length and number of which is sensitive to the value of the threshold used for detection: the lower the threshold, the more jumps are detected (some of which are FPs) and the shorter these partitions are, which, as explained below, can skew the estimate of *D*. A high threshold, on the other hand, leaves a significant number of true jumps in those segments. The goal here is thus to vary the threshold *θ* in order to obtain the optimal estimate of *D*.

In the limit of an infinitesimally small sampling interval, $\Delta t \rightarrow 0$, the quadratic variation $[Y^{\mathrm{diff}}(t)]$ of a pure diffusion process converges to the so-called integrated variation, which, for additive and time-independent noise, gives [40, 41]

20 $$ \bigl[Y^{\mathrm{diff}}(t)\bigr] = \int _{0}^{T}2D \, \mathrm{d}s = 2DT, $$

where $T = (n-1)\Delta t$ is the total duration for the *n* samples of $\{X(t)\}$. We can, therefore, estimate *D* via the sample quadratic variation, also known as realized variance, $RV(t)$ [40, 41]:

21 $$ \hat{D} \approx \frac{1}{2T} RV(t) = \frac{1}{2T} \sum_{k=0}^{n-1} \bigl( X^{\mathrm{diff}}(t_{k+1}) - X^{\mathrm{diff}}(t_{k}) \bigr) ^{2} . $$

For instance, for a test diffusive process with $F(y)=-\mathrm{0.2}y$ and $D=\mathrm{0.15}$ and sampled at $\Delta t = \mathrm{0.01}\mbox{ s}$ ($N=10^{6}$), Eq. (21) estimates *D* with an error of 0.002%. In contrast, applying Eq. (21) to a realization of a jump-diffusion process returns an overestimated *D̂*, as expected due to large, non-diffusive, positive increments that populate $\{X(t)\}$. Also note that if the threshold is set low enough to detect the smallest true jumps, in general, it can also remove the largest diffusive increments, which are required to properly apply Eq. (21). Simply removing the detected jumps from the sum in Eq. (21) would, therefore, yield an underestimated *D̂*.

To circumvent this problem, we consider only the negative increments of $\{X(t)\}$ in the calculation of *D̂*, as they will remain essentially unaffected by the presence of positive jumps, with the exception of a short transient following the jump offset. Indeed, following each jump, we expect to see a brief period where the process is out of equilibrium. And since the jumps have positive amplitudes, negative increment statistics are biased toward negative values during this transient (e.g., Fig. 1). The calculation of *D̂* is thus based on applying Eq. (21) to jump-free segments of $\{X(t)\}$, but only including negative increments, and neglecting the initial transient at the start of each segment (the duration of which is determined below). This is repeated for various values of *θ*. The successful estimation of *D* based only on negative increments relies on our assumption that Δ*t* is small, for in this case increments are approximately independent and distributed as $\mathcal{N}(0,2D\Delta t)$. For larger Δ*t*, the increment PDF can become asymmetric, meaning that the statistics of negative increments differ from those of positive ones, which would cause errors in our estimation of *D*.

Let $T_{\mathrm{off}}$ and $T_{\mathrm{on}}$ denote the jump offset and onset times, respectively. Note that the values of these times and the number of detected jumps all depend on the specific value of the threshold. Then the *i*th segment is defined by $\{S(t)\}_{i} = \{X(t) : T_{\mathrm{off}}(i) < t < T_{\mathrm{on}}(i+1)\}$ and is of duration $T_{i} = T_{\mathrm{on}}(i+1) - T_{\mathrm{off}}(i) $, and let $\{\Delta S(t)\}_{i}$ be its $n_{i}$ increments. Out of these $n_{i}$ increments, we keep only the $n_{i}^{-}$ that are negative and that occur after the transient of approximate duration *Φ*. We are thus left with the following subset of increments from each segment:

22 $$ \bigl\{ \Delta S(t) \bigr\} _{i}^{-} = \bigl\{ \bigl\{ \Delta S(t) \bigr\} _{i} : \bigl\{ \Delta S(t) \bigr\} _{i} < 0 , t > \varPhi \bigr\} . $$

For each segment, we obtain an estimate $\hat{D}_{i}$ as follows:

23 $$ \hat{D}_{i} = \frac{1}{2T_{i}^{-}} \sum _{k=1}^{n_{i}^{-}} \bigl( \bigl\{ \Delta S(t_{k})\bigr\} ^{-}_{i} \bigr)^{2}, $$

where $T_{i}^{-} = n_{i}^{-} \Delta t $ is the effective duration of the combined $n_{i}^{-}$ negative increments. We then calculate *D̂* as an average of the $\hat{D}_{i}$’s, weighted by $T_{i}/T$.

**Figure 5:**
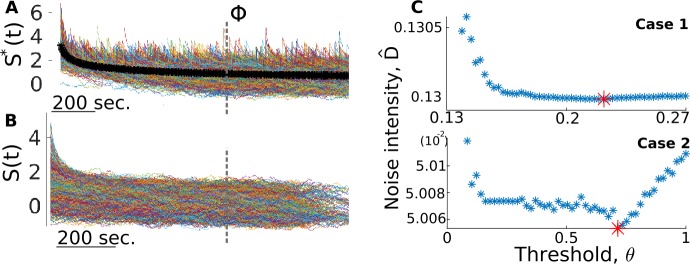
*Calculating* *D̂* *relies on partitioning* $\{X(t)\}$ *in jump-free segments for different threshold values*. We show here how this strategy is applied to the two jump-diffusion test cases presented in Sect. 3.2. (**A**) A jump-triggered average (black curve) is obtained from the largest 5% jumps in the data and is used to obtain a maximum estimate of the transient time scale *Φ* (dashed line). (**B**) Jump-free segments used to calculated the optimal value of *D̂* in C. (**C**) Different estimates of *D* are produced for different values of the threshold. We heuristically choose the minimum value as the optimal value. Traces in A and B are from Case 2
- Identify *Φ* as the approximate moment when the jump-triggered average stabilizes, quantified here as when its derivative is less than 0.05 (results do not depend strongly on this particular value: changing *Φ* by one order of magnitude on either side of the value used here yields estimates of *D* that differ by less than 0.2%). This gives us an estimate for the maximum time scale required for post-jump equilibrium.
- With *Φ* determined, and for each value of the threshold, extract the $\{S(t)\}_{i}$’s (Fig. 5(B)) and apply Eq. (23) to their negative increments for which $t > \varPhi $ (neglecting segments for which $T_{i}<\varPhi $).

More precisely, here are the steps taken in order to arrive at *D̂*:

- Starting from the largest value of $\{\Delta X(t)\}$, lower the threshold until the largest 5% of jumps are detected, which are the ones with the most prominent transient.
- Let $\{S^{*}(t)\}^{i}$ be the segments that follow these jumps (Fig. 5(A)). Average across them for each time step, creating a jump-triggered average trace (Fig. 5(A)), black line).

This scheme results in an estimate *D̂* for each value of the threshold, and the lowest value is chosen as the optimal estimate (Fig. 5(C)). This is because this approach overestimates *D* on both ends of the range of threshold values, but for different reasons. For low *θ*, many more jumps are detected, and this makes the $\{S(t)\}_{i}$’s shorter, which means that the associated *D̂* is more prone to be biased by the residual of the transient. On the other hand, a very high threshold leaves significant jumps in the $\{S(t)\}_{i}$’s, which biases the statistics of negative increments. The optimal balance is reached somewhere in-between, where the segments are large enough so that the initial transient is negligible, and where the jumps that are inevitably left in the segments do not significantly alter the statistics of negative increments. The heuristic choice of an intermediate value, namely one that corresponds to the minimal *D̂* estimate, gives excellent results in both validation cases (less than 0.1% error, see Table 2).

#### Drift function

Our estimation of the drift function *F* relies on the differential Chapman–Kolmogorov equation [42], which describes the evolution of the transition probability of a stochastic process where jumps occur alongside diffusive fluctuations. Let $Y(t)$ be a jump-diffusion process with transition probability $P_{Y|Y_{0}}$. For the case of positive Poisson jumps and additive diffusive noise, the differential Chapman–Kolmogorov equation reduces to (see the Appendix)

24 $$\begin{aligned} \frac{\partial P_{Y|Y_{0}}(y,t|y_{0},t_{0})}{\partial t} =& -\frac{\partial }{\partial y} \bigl[ F(y) P_{Y|Y_{0}}(y,t|y_{0},t_{0}) \bigr] + D \frac{\partial ^{2}}{\partial y^{2}} P_{Y|Y_{0}}(y,t|y_{0},t_{0}) \\ &{}- \lambda P_{Y|Y_{0}}(y,t|y_{0},t_{0}) + \lambda \int _{0}^{\infty } Q_{B}(s)P_{Y|Y_{0}}(y-s,t|y_{0},t_{0}) \,\mathrm{d}s. \end{aligned}$$

If $Y(t)$ is assumed to have reached its equilibrium state, then the left-hand side vanishes, and in the right-hand side we can replace the transition probability with the first-order equilibrium PDF, $P_{Y}$,^2^ which we assume to be equal to the empirical PDF, $P_{X}$, of the measured time series $\{X(t)\}$. The drift function can then be evaluated from Eq. (24) if *D*, *λ*, and $Q_{B}$ are known (or estimated).

Note, however, that *F̂* is required in the first step of branch III of the iterative procedure (Fig. 4), since the FP-related statistics, $\varGamma _{A}$ and $Q_{A}$, are calculated based on the drift function. A preliminary estimate $\hat{F}_{1}$ of the drift function is thus required. This particular estimate, which is needed only once throughout the inference procedure, is obtained by letting $\lambda = 0$ in Eq. (24), such that it becomes the Fokker–Planck equation associated with the diffusive part of the stochastic process. The stationary solution of this Fokker–Planck equation can be used to establish a relation between the noise intensity, the drift function, and $P_{Y}$ [37, 43]:

25 $$ P_{Y}(y) = \frac{K}{\hat{D}} \exp \biggl( - \int \frac{\hat{F}_{1}(y)}{ \hat{D}} \,\mathrm{d}y \biggr), $$

where *K* is a normalization constant and where, again, we assume that $P_{Y}=P_{X}$. This first preliminary estimate is necessarily flatter than the true *F*, as the presence of jumps makes $P_{X}$ wider than it would be if there were no jumps. Successive iterations gradually rectify this by incorporating estimates of *λ* and $Q_{B}$ in Eq. (24).

## Results

Here we present three applications of the method developed above. First, we validate the calculation of $\varGamma _{A}$ and $Q_{A}$ for the case of a purely diffusive process. Then we apply the full iterative scheme to two simulated jump-diffusion processes with different characteristics. Finally, we apply our inference method to electrophysiological recordings in pyramidal cells of electric fish.

### Validation of the FP statistics calculations

To confirm that the calculations of $Q_{A}$ and $\varGamma _{A}$ are accurate, we start with a simple test case where we consider a time series $\{X^{\mathrm{diff}}(t)\}$ obtained from a simulated pure diffusion process. As there are no jumps here, the distribution of increments does not possess the necessary asymmetry to properly identify a threshold. For this test case only, we thus opt for a specific value, $\theta ^{*}=0.1$, that showcases the ability of our method to handle FPs of various durations. The results presented here, however, remain valid for a range of values of $\theta ^{*}$. For the parameters used in this pure diffusion validation case (Table 1), this range extends from 0.025 up to 0.2. The upper limit is set by the fact that, beyond it, too few FPs are detected, which precludes any statistical calculations from being achieved. The lower limit, on the other hand, arises because too many FPs are detected, such that, for instance, they occur every other time step. In such a case, Δ*t* is too large and the estimation of *λ* becomes imprecise due to the statistical fluctuations in the number of detected FPs.

Applying the threshold in this case leads to a detected jump pool comprised entirely of FPs. The goal now is to compare the measured $Q_{C}$ and $\varGamma _{C}$ with the calculated $Q_{A}$ and $\varGamma _{A}$. If we obtain that $\varGamma _{C} \approx \varGamma _{A}$ and that $Q_{C} \approx Q_{A}$, then we will effectively have shown that the true jump rate is zero, $\lambda = 0$, and that our method correctly calculates the FP amplitude distribution. In this pure diffusion test case, these calculations rely on the knowledge of the correct noise intensity *D* and the correct drift function *F*, but this will not be the case in subsequent sections.

**Figure 6:**
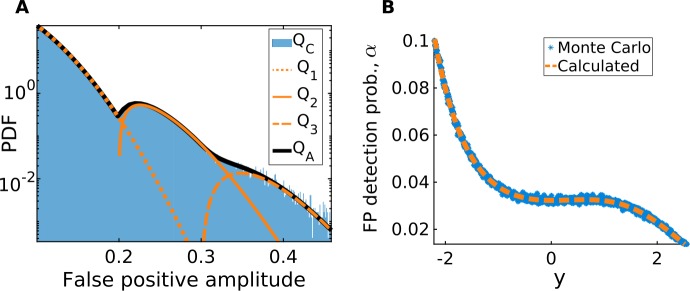
*For a pure diffusion process*, *we correctly calculate the FP amplitude distribution* $Q_{A}$ *and FP detection probability* $\varGamma _{A}$. (**A**) After applying a threshold to $\Delta X^{\mathrm{diff}}(t)$, we obtain a pool of FPs with a range of durations and amplitudes. From the latter we measure $Q_{C}$ (blue histogram). We correctly calculate this distribution as $\sum_{i}Q_{i}$, where $Q_{i}$ is defined in Eq. (26). (**B**) Probability of detecting an FP as a function of *y*, calculated (yellow curve) and Monte Carlo simulated (blue dots)

With the particular values of *D* and *F* used here to simulate $\{X^{\mathrm{diff}}(t)\}$ (Table 1), we find that FPs are either singlets, doublets, or triplets, which contribute differently to the measured amplitude distribution $Q_{C}$. Indeed, the fact that longer FPs tend to have larger amplitudes and that FP durations are always multiples of Δ*t* creates distinct modes in the measured amplitude distribution (Fig. 6(A), blue histogram). By taking the sum out of the integral in Eq. (9), we can write $Q_{A}(a) = \sum_{i=1}^{\infty }Q_{i}(a)$, where

26 $$ Q_{i}(a) = \int _{-\infty }^{\infty }P_{A|\tau ,Y_{0}}(a|i\Delta t,y) P_{\tau |Y_{0}}(i\Delta t|y) P_{Y_{0}}(y) \,\mathrm{d}y $$

are the individual distributions associated with FPs of duration $i\Delta t$ (Fig. 6(A), yellow curves). These distributions are then summed to obtain $Q_{A}$, which is a precise match with $Q_{C}$ for this purely diffusive example (Fig. 6(A), black curve).

Furthermore, by applying Eq. (5) we calculate the *y*-dependent detection probability, $\alpha (y)$ (Fig. 6(B), yellow curve). As expected, this function depends non-trivially on *y* and reflects the nonlinearity of the specific drift function used in this example. If multiplicative noise had been used, the noise intensity $D(y)$ would also have influenced the shape of $\alpha (y)$. To validate this calculation of $\alpha (y)$, we run Monte Carlo simulations of the diffusion process

27 $$ dY^{\mathrm{diff}}(t) = F\bigl(Y^{\mathrm{diff}}(t)\bigr)\,dt + \sqrt{2D} \,dW(t), $$

over a duration Δ*t*, but with a time step of $\Delta t/1000$ and with various initial conditions along the *y*-axis. For each initial condition, we evaluate the FP detection probability as the ratio between the number of Monte Carlo runs, where $Y^{\mathrm{diff}}(\Delta t) > Y ^{\mathrm{diff}}(0) + \theta ^{*}$, and the total number of Monte Carlo runs (Fig. 6(B), blue dots), the result of which precisely fits with the calculated $\alpha (y)$. Finally, we obtain the overall FP detection probability $\varGamma _{A}$ from Eq. (6), which, in this pure diffusion test case, differs from $\varGamma _{C}$ by only 0.06%.

### Validation of the iterative scheme

**Figure 7:**
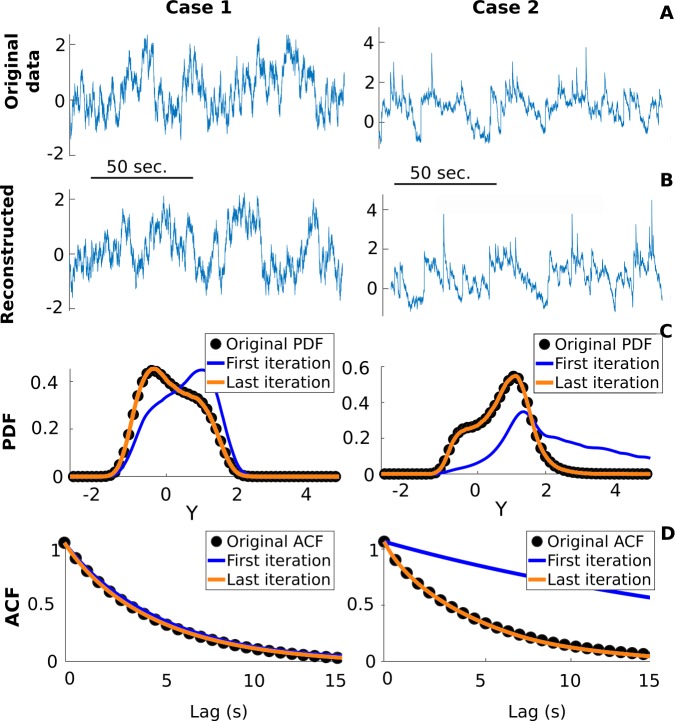
*Our inference method successfully fits a stochastic model to the original data*. (**A**) Realizations of the jump-diffusion processes for the two different validation cases. (**B**) Simulation results from the jump-diffusion SDE inferred by our method. (**C**) Comparison between the PDF of the original validation data (black dots) and that of the last iteration of the fitted SDE (yellow curve). The PDF associated with the first iteration is also shown (blue curve). (**D**) Similar comparison between the original and estimated ACF

Before applying the iterative procedure (Fig. 4) to real data, we first validate it against time series generated by numerically integrating Eq. (1) (using the Euler–Maruyama scheme). We consider two validation cases: in Case 1 the amplitude of the jumps is comparable to the diffusive fluctuations and jumps are sparser in time (i.e., occur at a lower rate) than in Case 2, where jumps are much larger than the background noise and their rate is double that of Case 1 (Fig. 7(A)). The specific functions and parameters used to generate and analyze these validation data are shown in Table 1, which can be summarized as follows: low rate, low amplitude, high noise for Case 1, and high rate, high amplitude, low noise for Case 2. Preliminary tests with a linear drift function showed a successful fit between the fitted SDE and the numerical data. We now opt for a more general and arbitrary shape where the drift function is nonlinear and non-monotonic. The only restrictions are that it yields a single stable fixed point and that the resulting stochastic process is stationary. We thus restrict our analyses to drift functions that are mostly decreasing. Although the parameters and functions used for the simulations are known, they are not used in the inference procedure, only $\{X(t)\}$ is. To assess the performance of the proposed method, we compare the estimated *D̂*, *λ̂*, *F̂*, and $\hat{Q}_{B}$ with their true values.

**Table 1:** Parameters and functions for the validation cases

	Case 1	Case 2	Pure diffusion
*λ*	0.1	0.2	0
*D*	0.13	0.05	0.15
$\theta ^{*}$	0.125	0.07	0.1
*μ*	−1.2	1	N/A
*σ*	0.2	0.5	N/A
$Q_{B}$	$\mbox{Lognormal}(\mu ,\sigma ^{2})$		N/A
*a*	0.2	0.2	0.2
*F*	$-(a(y-0.5)^{3} +0.5a(y-0.7)^{2} + 0.1)$		
Δ*t* (s)	10^−2^	10^−2^	10^−2^

We choose these two specific cases because they challenge both the sensitivity and the robustness of our method. In Case 1, the statistics of $\{X(t)\}$ are not too different from those of a pure diffusion process, which makes it easier to estimate *F*, but difficult to extract $Q_{B}$ amidst the FPs. In contrast, the jumps in Case 2 are well separated from the diffusive fluctuations, which allows for a more direct estimation of $Q_{B}$. The presence of large jumps in this case, however, significantly alters $\{X(t)\}$ and its PDF, making it harder to estimate *F*. In both cases, however, we find that the original SDEs can be precisely recovered by our method. For instance, simulating Eq. (1) with *D̂*, *λ̂*, *F̂*, and $\hat{Q}_{B}$ of the last iteration not only produces time series that resemble the originals (Figs. 7(A) and 7(B)), but also yields an excellent fit between the reconstructed and original PDFs (Fig. 7(C))), with an $O(10^{-4})$ root-mean-square error (normalized by the range of $\{X(t)\}$) and ACFs (Fig. 7(D)).

**Figure 8:**
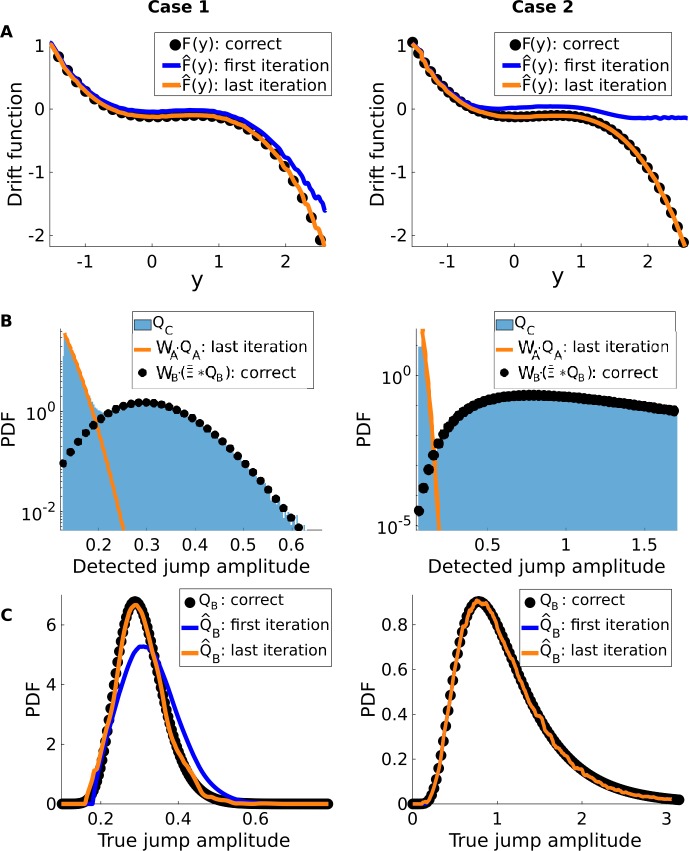
*Our inference procedure recovers the correct parameters and functions from Eq.* (1). (**A**) The correct drift function (black dots) is recovered in the last iteration (yellow curve). First iteration results are shown for comparison (blue curves). (**B**) We see that the measured amplitude distribution $Q_{C}$ is in fact a mixture of $Q_{A}$ (yellow curve) and $\tilde{\varXi }\ast Q_{B}$ (black dots, see Eq. (19)). (**C**) After deconvolving the latter, we do recover the correct $Q_{B}$ (yellow curve). Note that in Case 2, the first and last iterations are confounded, as the true jump amplitudes are almost directly separable from those of FPs

Inspecting the results from the last iteration, we indeed see that the correct drift function is recovered (Fig. 8(A)). This is done through the use of the so-called differential Chapman–Kolmogorov equation. This is then used to calculate the next $Q_{A}$ and $\varGamma _{A}$ (Fig. 8(B)), which allows the correct $Q_{B}$ to be demixed from the measured $Q_{C}$ (Fig. 8(C)). We also obtain low relative errors when comparing our estimates and the correct values of *D* and *λ* (Table 2). Note also that, although not shown here, we obtain the same fit quality between original and reconstructed when we apply our method to hybrid cases, where, for instance, the small jump amplitudes are paired with a higher rate instead of a lower one and vice-versa.

**Table 2:** Comparison between estimated and correct parameters

	Case 1		
	Correct	Estimated	Relative error (%)
D	0.13000	0.13003	0.023
*λ*	0.1000	0.0978	2.2

	Case 2		
	Correct	Estimated	Relative error (%)
D	0.05000	0.05005	0.1
*λ*	0.2000	0.1960	2.13

### Application to experimental data

We now proceed with an application of our inference method to electrophysiological data published in Ref. [44]. These data consist of in vitro, intracellular recordings of membrane voltage fluctuations in pyramidal neurons of the weakly electric fish *Apteronotus leptorhynchus*. These fish are endowed with an active sensing mechanism whereby they generate a high frequency (∼700 to 1000 Hz) oscillatory electric field around their body. This electric signal, along with any distortions caused by objects, preys, and conspecifics, is sensed by electroreceptors located on the fish’s body. This information is then sent to the hindbrain, where it reaches the first stage of electrosensory processing, called the electrosensory lateral line lobe (ELL). The recordings in Ref. [44] were taken from neurons of the ELL, specifically in the centrolateral and centromedial segments (CLS and CMS, respectively). In order to isolate the impact of voltage-gated ion channels on membrane potential fluctuations, the ELL was treated with pharmacological agents (CNQX and APV) that block synaptic transmission onto the pyramidal cells. The resulting fluctuations are thus fully attributed to cell-intrinsic sources, which we refer to as membrane noise. The main source of this type of noise is often assumed to be the stochastic opening and closing of ion channels, i.e., channel noise [45]. In this case, we cannot rule out other potential contributions, as non-trivial soma-dendrite interactions have been observed in these cells [46]. Note also that, by imposing different holding currents on the cells, Ref. [44] recorded ongoing membrane noise at various levels of hyperpolarization relative to spike threshold.

**Figure 9:**
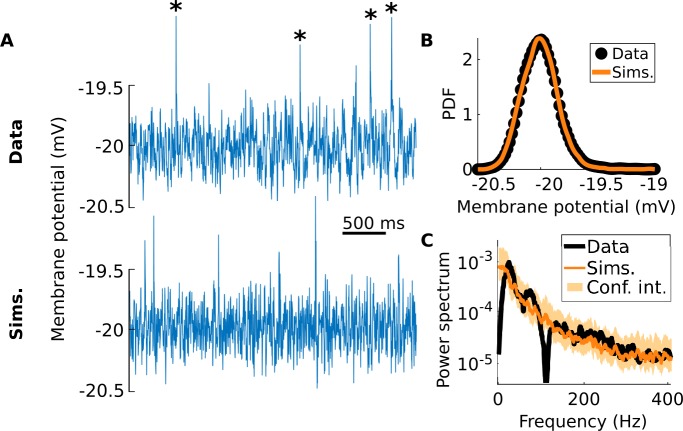
*Membrane noise in CLS cells can be modeled with an jump-diffusion SDE*. (**A**) An exemplar recording of membrane voltage fluctuations in a CLS pyramidal neuron (top). A holding current is used to maintain the cell at 20 mV below its spike threshold. Asterisks show the four largest blips found in this trace. Under the assumptions listed in Sect. 2.1, simulations of the fitted SDE (bottom) are qualitatively similar to the original data (here $\Delta t = 1.2\text{ ms}$). (**B**) This similarity is confirmed by the close match between the data and simulation PDFs (yellow curve and black dots, respectively). (**C**) Despite having no role to play in the inference procedure, the power spectrum of the data (black) also fits with that of the simulations (yellow). The notches in the power spectrum of the data result from the removal of experimental artifacts

Of interest here is the presence of large, jump-like events, called blips, that abruptly depolarize the cells (Fig. 9(A), asterisks). Although Ref. [44] puts forth a hypothesis as to the functional role for these blips, the mechanism underlying their occurrence is unknown. This, along with the limited amount of data, hinders the development of any meaningful mechanistic model of this phenomenon. The jump-diffusion inference approach developed here, however, is particularly well suited to circumvent this knowledge gap. Indeed, the resulting phenomenological models provide a useful tool for dynamically interpreting the available data without relying on poorly constrained biophysical mechanisms. For instance, we can address questions such as: Do certain parameters or functions of the model change as a function of the mean membrane potential?

Here we analyze recordings from two CMS and two CLS cells, each with five or six levels of hyperpolarization: from −25 to 0 mV below threshold, with 5 mV steps between levels. Using these relative levels with respect to spike threshold is required to compare cells that might have different thresholds (e.g., −67 to $-63~\mbox{mV}$ for CMS cells [44]). After removing experimental artifacts (see Sect. 3.4), we obtain a total of 23 traces, each lasting approximately 10 s. Applying our inference method to these traces yields a good fit between the resulting simulations and the original data (Fig. 9(A)): the PDFs differ only by $O(10^{-2})$ normalized root-mean-square errors, and the power spectra fall within 95% confidence intervals of each other (Figs. 9(B) and 9(C)).

**Figure 10:**
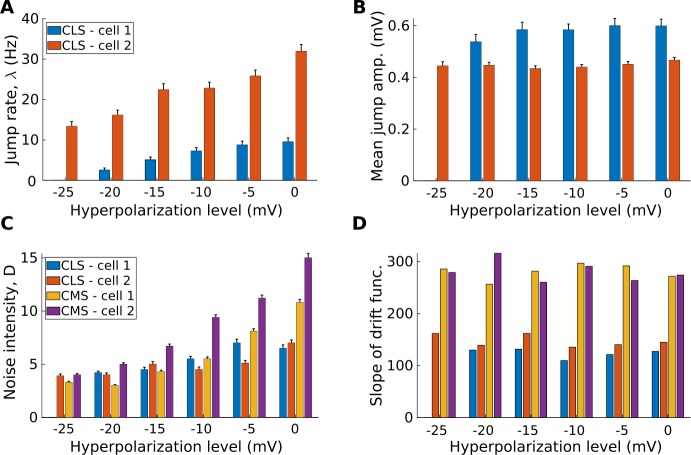
*CLS cells increase their rate and noise intensity*, *but not jump amplitudes*, *when they approach threshold*, *and all cells maintain a steady drift function across levels*. (**A**) Jump rate of CLS cells. Error bars show one standard deviation, assuming that the number of detected blips is Poisson. (**B**) Mean amplitudes of the blips. Error bars show one standard deviation, calculated from 1000 bootstrap samples of the original amplitude values. We observe a similar lack of systematic trend in the variance of the amplitudes (not shown). (**C**) Noise intensity for all cells. Error bars show one standard deviation, calculated from 1000 bootstrap samples of the original data increments. (**D**) Local slope of the drift function, as determined from a linear fit over a ${\pm}2~\mbox{mV}$ range around the stable fixed point. Error bars are too small to see on this scale, but are calculated as 95% confidence interval of the slope parameter of the linear least square fit

Further insight can be gained by comparing the estimated SDE parameters and functions *D̂*, *λ̂*, $\hat{Q}_{B}$, and *F̂* across all traces. We thus see, for instance, that CLS cells increase their jump rate (Fig. 10(A)), but not jump amplitudes (Fig. 10(B)), as they approach threshold. Note also that we could not measure any significant jump component for CMS cells. Instead, fluctuations in these cells are well described by pure diffusion. Furthermore, all cells show an increase in their diffusive noise intensity with depolarization, and this is more prominent in CMS cells (Fig. 10(C)). Lastly, to compare the different drift functions with a scalar measure, we apply a linear fit to *F* (estimated as in the previous section) in the vicinity (${\pm}0.2~\mbox{mV}$) of the stable fixed point. The slope parameter resulting from this fit can be interpreted as a measure of how wide or narrow the potential function is around the resting membrane voltage. Using this measure, we find no systematic intra-cell trend, but we do observe large differences between cell types: CLS cells have a wider potential function than CMS ones (Fig. 10(D)).

### Data processing

For each cell, the raw data consist of a continuous, 60 to 70 second staircase-like trace, sampled at 20 kHz. Each step lasts ∼10 s and corresponds to a different holding current, which was applied such as to create 5 mV hyperpolarization from the previous level. In order to segment the recordings into different traces for each level, we first identify the transition times between different holding currents. This is done visually, as the transitions are unambiguous, and we omit ±0.5 seconds around those times. At the −5 and $0~\mbox{mV}$ level, a few spikes (1 to 4) occur in the recordings. They are manually removed from the traces along with the ensuing refractory period.

A conspicuous aspect of the resulting traces is the presence of slow, large amplitude (1 Hz, ${\sim}1~\mbox{mV}$) quasi-oscillations overlaid with the faster, stochastic fluctuations. The exact source of this slow component is unknown, but is possibly related to persistent sodium channels, which have been shown to populate the soma and proximal apical dendrites of ELL cells [44] and to produce slow perithreshold oscillations in entorhinal stellate neurons [47]. In any case, these slow oscillations are outside the scope of the method presented here. A moving average filter (0.05 s window size) is thus applied to remove this low frequency content from the signal.

Line noise is removed at all 60 Hz multiples with a notch filter, but the data are also contaminated with artifacts in other frequency bands, potentially from interference with other sources. This is most prominent in the 900–3000 Hz, but also carries around lower frequencies, e.g., 100, 270, and 550 Hz. To account for this artifact, we opt for the combination of a low-pass filter with a 900 Hz cut-off, and 20 Hz wide band-stop filters centered on the other problematic frequencies. Electrophysiological recordings can be dominated by measurement noise at high frequencies [48]. In our case this is seen as a flattening out of the PSD above 1000 Hz, so the 900 Hz cut-off used here does not lead to the loss of important biological signals.

The end result of this processing chain are time series that exhibit fluctuations typical of jump-diffusion processes. We do observe, however, significant higher-order correlations on the smallest timescales ($\mathrm{O}(\Delta t)$, $\Delta t = 50~{\mu}\mbox{s}$). To quantify these correlations, we use the notion of the Einstein–Markov timescale [5]. This is a measure of the timescale below which the Markov property no longer holds. Stochastic time series often show a departure from the Markov property on small timescales, possibly due to noise source correlation, the presence of an inertial component in the dynamics, or measurement noise [5]. Following [5] and [49], we estimate this Einstein–Markov timescale by finding the value of *τ* that minimizes

28 $$ \chi ^{2} = \iiint \frac{ [ P(x_{1},x_{2},x_{3}) - P(x _{3}|x_{2})P(x_{2},x_{1}) ]^{2}}{\sigma ^{2}}\,\mathrm{d}x_{1} \, \mathrm{d}x_{2}\,\mathrm{d}x_{3}, $$

where $x_{1}=x(t)$, $x_{2}=x(t+\tau )$, $x_{3}=x(t+2\tau )$, and $\sigma ^{2}$ is the sum of the traces of the covariance matrices associated with the distributions in the numerator. For a proper Markov process, $\chi ^{2}=0$, ∀*τ*. In this case, we find the minimum of $\chi ^{2}$ at 1.2 ms, indicating that the Einstein–Markov timescale of the data is over one order of magnitude larger than the sampling interval Δ*t*. This means that, on the time scale of individual observations, the data evolve with a history dependence that is incompatible with a Markovian description. If, however, we look at the data on a coarser time scale, e.g., the Markov–Einstein time scale of 1.2 ms, then the Markov property is approximately satisfied. In that case only can we hope to use Eq. (1) as a valid model for these data. To account for this problem, we resample the data at a 1.2 ms interval (∼830 Hz sampling rate) and obtain the final time series on which to apply our method (Fig. 9(A), top), with the time step equal to the Markov–Einstein time scale. Note that this situation is conceptually similar to how the Langevin model of diffusion (where the position of a particle is, by itself, not Markovian) reduces to the Einstein model (where the position is Markovian) only above a certain time scale [43].

## Discussion

In this study, we develop an iterative procedure that recovers the parameters and functions of a jump-diffusion SDE, based solely on a realization of the associated stochastic process. This approach is validated when the jumps are comparable in size to the diffusive fluctuations (Case 1), as well as when they are much larger than diffusive fluctuations (Case 2). We apply this method to membrane voltage fluctuations recorded in pyramidal neurons of electric fish. Our analysis reveals that these data can indeed be represented as jump-diffusion processes. We find that pyramidal neurons increase their jump rate and noise intensity as they approach spike threshold, while their jump amplitudes and drift function remain unchanged.

Our approach relies on five main components: the use of the differential Chapman–Kolmogorov equation to estimate the drift function, the use of quadratic variation on jump-free segments to estimate the diffusive noise intensity, the detection of jumps via threshold-crossing of the increments, the modeling of detected jumps as a mixture of true jumps and FPs, and the calculation of FP statistics used to extract true jump statistics from the detected jump pool.

Although we estimate the drift function and the true jump amplitude distribution non-parametrically, we do limit our study to the case of additive diffusive noise, of constant jump rate, and of Poisson jumps. Relaxing the additive noise assumption would require an estimation scheme for the diffusion function $D(y)$. For purely diffusive processes, this function can be obtained directly through the estimation of the second Kramers–Moyal coefficient, which is defined in terms of the second conditional moment of the increments. Evaluating this moment simply requires the knowledge of the conditional PDF across time steps. For a Poisson jump-diffusion process, however, Ref. [31] has shown that the diffusion function can in fact be expressed in terms of the second conditional moment of the increments, the jump rate, and the second moment of the jump amplitudes. It should thus be possible to include the estimation of $D(y)$ into the iterative portion of our method (Fig. 4). Indeed, estimates of the jump rate and of the amplitude distributions could be used at each pass to estimate the diffusion function. Furthermore, we have limited our analysis to noise intensities for which jump amplitudes are on average an order of magnitude or more larger than diffusive fluctuations. When diffusive fluctuations and jumps are of similar average magnitude, the number of detected FPs becomes too large and estimates of *λ* and $Q_{A}$ become imprecise due to increased statistical fluctuations. A much finer temporal resolution would be necessary to address this particular case.

As for the assumption of constant jump rate, it should be possible to extract a rate function $\lambda (y)$ as long as a *y*-dependent version of Eq. (17) can be written. This would require a long enough data time series such as to produce an estimate of $\varGamma _{C}(y)$. Relaxing the assumption of Poisson jumps, however, would be more difficult to do. The detection probability of true jumps, $\varGamma _{B} = \lambda \Delta t$, would obviously need to be modified with the appropriate expression. Moreover, the specific form of the differential Chapman–Kolmogorov used here, Eq. (24), relies on the assumption of Poisson jumps (see the Appendix) and would thus need to be extended in a manner that depends on the precise non-Poissonian nature of the jump process. More specifically, the last two terms in Eq. (24), which are originally defined based on the transition rates of the Poisson jump process, would now be derived from the modified $\varGamma _{B}$. Note that, for the special case of true jumps with zero-mean amplitudes, the drift function can be estimated directly from the first conditional moment of the increments, without relying on Eq. (24) [31].

### Membrane noise

The unusual characteristics of membrane noise observed in CLS neuron, initially reported in Ref. [44] and represented here as jump-diffusion SDEs, might be implicated in novel ways in electrosensory processing. The analysis we perform here is a first step toward investigating this possibility computationally.

The positive impact of noise on information processing in neural systems is widely recognized [50]. Although channel noise was initially thought to be too weak compared to synaptic noise to influence a neuron’s output statistics [51, 52], it has since been shown to significantly impact neuronal reliability and action potential timing [47, 53–57]. Because it arises from the stochastic opening of voltage-gated ion channels, channel noise has been successfully modeled by populations of Markov chains with voltage-dependent transition rates [45]. In the quest for more computationally efficient models, however, various approximations have been used to model the collective behavior of these Markov chains as simple diffusion SDEs [57–60] (sometimes called the diffusion approximation for channel noise [61, 62], in reference to the diffusion approximation^3^ for synaptic bombardment). One of these approaches, for instance, introduces a current noise term directly in the membrane equation [57, 63], effectively modeling the subthreshold voltage locally as an Ornstein–Uhlenbeck process. It is perhaps not surprising then that we obtain a good match between the observed CMS membrane noise (at various holding potential) and a pure diffusion SDE. In cases where membrane noise is more accurately described by multiplicative conductance noise [57], however, we might expect deviation from the simple SDE used here, similar as to how the diffusion approximation can misrepresent the subthreshold voltage distribution for certain types of synaptic drive [64].

We cannot completely exclude the possibility that small, hard to detect blips occur in CMS cells. Although our method proves capable of handling this type of situation (Sect. 3.2), the limited amount of available experimental data in this case precludes us from conclusively ruling out the existence of a jump component in models of CMS membrane noise. In addition, in both types of cells we find a positive correlation between the noise intensity and the holding potential (Fig. 10). This is consistent with how membrane potential variance has been observed to increase with depolarization in these same cells [44], as well as in rat neocortical pyramidal neurons [65]. Simple Markov models involving only Na^+^ and K^+^ channels are able to reproduce this correlation [66].

The presence of blips in CLS neurons suggests that ion channels co-activate to produce abrupt depolarizing currents. It was shown in [67] that sodium channels appear in clusters, or hot spots, in ELL pyramidal cells. This might allow local depolarization of the membrane to sufficiently couple channels within a cluster. Alternatively, perhaps channels are physically and functionally coupled through scaffolding protein complexes, as observed in [68]. Regardless of the exact coupling mechanism, the commonly used assumption of independence between channels [45, 57] is likely violated by these blips. The fact that we have successfully fitted a jump-diffusion SDE to CLS membrane noise suggests that a diffusion-like approximation could be applied in this case as well. Such a deductive approach would, however, require tentative descriptions of local channel coupling to be included in the kinetic schemes.

Given the unknown biophysical mechanism underlying the blips, our fitted jump-diffusion model is uniquely positioned to address questions related to their functional role. Future work will thus aim to incorporate our fitted jump-diffusion model as a membrane noise term in a more complete model of CLS cells [69]. By accounting for the synaptic input associated with electrosensory input, the resulting model could investigate the possibility that blips assist or influence spiking, perhaps through a stochastic resonance-like phenomenon. Stochastic resonance, and more generally stochastic facilitation, has been shown to be mediated by channel noise in models of auditory brain stem neurons [51] and in modeled neuronal arrays [70], as well as to be mediated by synaptic noise in models of neocortical pyramidal neurons [71]. Since blips share a similar shape and amplitude as AMPA-driven excitatory post-synaptic potential, this begs the question of whether stochastic resonance is at play in the detection of weak electrosensory signals, such as small prey. This was indeed hypothesized, but not explicitly shown, in Ref. [44]: the voltage-dependence of membrane noise makes it impossible to vary the noise level, the rate of occurrence, or the amplitude of blips independently of the membrane potential. The value of this hypothesis, however, could be assessed by performing a numerical experiment with our fitted SDE representation of blip-laden membrane noise.

Lastly, although we apply our method here to recordings where synaptic input is completely blocked, it might be applicable to certain type of synaptic input patterns, such as correlated bombardment. We also hope to apply this method to fluctuations of the active-sensing rate of pulse-type electric fish, where jump-like events also occur [72].

## Appendix

The particular form of the differential Chapman–Kolmogorov equation that we use here, Eq. (24), relies on the assumption of Poisson jumps. For completeness, and to make explicit the dependence of this equation on Poisson statistics, we provide the following short derivation:

Let $X(t)$ be a continuous-time Markov process, possibly with discontinuous sample paths, in one dimension and with transition probability $p(x,t|x_{0},t_{0})$. The Chapman–Kolmogorov equation then has the following differential form [42]:

29 $$\begin{aligned} \frac{\partial p(x,t|x_{0},t_{0})}{\partial t} =& -\frac{\partial }{ \partial x} \bigl[ F(x,t) p(x,t|x_{0},t_{0}) \bigr] + \frac{ \partial ^{2}}{\partial x^{2}} \bigl[ D(x,t) p(x,t|x_{0},t_{0}) \bigr] \\ &{}+ \int \bigl[ W(x|\tilde{x},t)p(\tilde{x},t|x_{0},t_{0}) - W( \tilde{x}|x,t)p(x,t|x_{0},t_{0}) \bigr] \, \mathrm{d}\tilde{x}, \end{aligned}$$

where

30 $$ W(x|\tilde{x},t) \equiv \lim_{\Delta t \rightarrow 0} \frac{p(x,t+ \Delta t|\tilde{x},t)}{\Delta t}. $$

The first and second term of Eq. (29) are associated with the continuous part of $X(t)$ and are often referred to as the advective and diffusive terms in the parlance of partial differential equations [38]. The last term in Eq. (29), on the other hand, represents how probability mass is transferred through discontinuities in $X(t)$, as the function *W* is the instantaneous transition rate between different values of *x*. Indeed, sample path continuity is expressed as $W(x|\tilde{x},t) = 0$ [42], and in that case, Eq. (29) reduces to the Fokker–Planck equation. Note that, in the case where both the drift and diffusion functions vanish, Eq. (29) reduces to the so-called Master equation in statistical mechanics [36, 73].

In order to evaluate Eq. (30), we first introduce three mutually exclusive and collectively exhaustive events: $E_{0}=\{\mbox{no jumps in }[t,t+\Delta t]\}$, $E_{1}=\{\mbox{exactly one jump in }[t,t+ \Delta t]\}$, and $E_{2}=\{\mbox{two or more jumps in }[t,t+\Delta t]\}$, such that $E_{i} \cap E_{j} = \varnothing $, for $i \neq j$, and $\operatorname{Prob} ( \bigcup_{i} E_{i} ) =1$. From the law of total probability, we get

31 $$ p(x,t+\Delta t|\tilde{x},t) = \sum _{i=1}^{3} p(x,t+\Delta t|\tilde{x},t;E _{i}) \cdot \operatorname{Prob}(E_{i}). $$

Assuming that discontinuities occur through a Poisson process with parameter *λ*, we can use the well-known results [74, 75]:

32 $$ \begin{aligned} &\operatorname{Prob}(E_{0}) = 1 - \lambda \Delta t + o(\Delta t), \\ &\operatorname{Prob}(E_{1}) = \lambda \Delta t + o(\Delta t), \\ &\operatorname{Prob}(E_{2}) = o(\Delta t), \end{aligned} $$

where $o(\Delta t)$ goes to zero faster than Δ*t*:

33 $$ \lim_{\Delta t \rightarrow 0}\frac{o(\Delta t)}{\Delta t} = 0. $$

Inserting the sum in Eq. (31) into the limit of Eq. (30) results in the sum of three limits, associated with $E_{0}$, $E_{1}$, and $E_{2}$:

34 $$ W(x|\tilde{x},t) \equiv \lim_{\Delta t \rightarrow 0} \sum _{i=0}^{3} \frac{p(x,t+ \Delta t|\tilde{x},t; E_{i})\cdot \operatorname{Prob}(E_{i})}{\Delta t}, $$

where the first term vanishes because of sample path continuity between jump events $\lim_{\Delta t \rightarrow 0}p(x,t+\Delta t|\tilde{x},t;E _{0})/\Delta t=0$, while the third one vanishes as per Eq. (33) $\lim_{\Delta t \rightarrow 0}\operatorname{Prob}(E_{2})/\Delta t =0$. If jump amplitudes are distributed according to $Q_{B}$, then this distribution can be defined as $Q_{B} \equiv \lim_{\Delta t \rightarrow 0}p(x,t+ \Delta t|\tilde{x},t;E_{1})$, which results in

35 $$\begin{aligned} W(x|\tilde{x},t) &= \lim_{\Delta t \rightarrow 0}\frac{p(x,t+\Delta t| \tilde{x},t,E_{1})\cdot \operatorname{Prob}(E_{1})}{\Delta t} \\ &= \lim_{\Delta t \rightarrow 0}\frac{p(x,t+\Delta t|\tilde{x},t,E _{1})\cdot (\lambda \Delta t + o(\Delta t))}{\Delta t} \\ &= \lambda Q_{B}(x-\tilde{x}). \end{aligned}$$

When inserted into Eq. (29), this yields

36 $$\begin{aligned} \frac{\partial p(x,t|x_{0},t_{0})}{\partial t} =& -\frac{\partial }{ \partial x} \bigl[ F(x,t) p(x,t|x_{0},t_{0}) \bigr] + \frac{ \partial ^{2}}{\partial x^{2}} \bigl[ D(x,t) p(x,t|x_{0},t_{0}) \bigr] \\ &{}- \lambda p(x,t|x_{0},t_{0}) + \lambda \int Q_{B}(x-\tilde{x})p( \tilde{x},t|x_{0},t_{0}) \,\mathrm{d}\tilde{x}. \end{aligned}$$

If we assume a time-homogeneous drift function, additive noise, and a positive support for $Q_{B}$, we recover Eq. (24).

## Footnotes

1 Alternatively, one could obtain Eq. (9) directly from Eq. (7) by invoking the law of total probability [74] for the set of mutually exclusive and exhaustive events $\{\tau = i\Delta t, i \in \mathbb{N}^{*}\}$.

2 This equilibrium PDF exists for stable processes and is defined by $P_{Y}(y) = \lim_{t-t_{0} \rightarrow \infty } P_{Y|Y_{0}}(y,t|y_{0},t_{0})$ [43].

3 In this case we refer to how stochastic synaptic currents, modeled as shot noise and assumed to arrive at a high rate, can be approximated as a diffusion process.

## Abbreviations

SDE: stochastic differential equation
PDF: probability density function
ACF: autocorrelation function
PSD: power spectral density
FP: false positive
ELL: electrosensory lateral line lobe
CLS: centrolateral segment
CMS: centromedial segment
